# Mucosal response of inactivated and recombinant COVID‐19 vaccines in Congolese individuals

**DOI:** 10.1002/iid3.1116

**Published:** 2023-12-27

**Authors:** Freisnel H. Mouzinga, Constanze Heinzel, Abel Lissom, Andrea Kreidenweiss, Armel L. Batchi‐Bouyou, Jacques D. Mbama Ntabi, Jean C. Djontu, Etienne Ngumbi, Peter G. Kremsner, Rolf Fendel, Francine Ntoumi

**Affiliations:** ^1^ Fondation Congolaise pour la Recherche Médicale Brazzaville Republic of Congo; ^2^ Faculté des Sciences et Techniques Université Marien Ngouabi Brazzaville Republic of Congo; ^3^ Institute of Tropical Medicine University of Tübingen Tübingen Germany; ^4^ Department of Zoology, Faculty of Science University of Bamenda Bamenda Cameroon; ^5^ Centre de Recherches Médicales de Lambaréné (CERMEL) Lambarene Gabon; ^6^ German Center for Infectious Diseases (DZIF) Partner Site Tübingen Tübingen Germany; ^7^ Global Clinical Scholars Research Training Program Harvard Medical School Boston Massachusetts USA

**Keywords:** antibody level, IgG, inactivated and recombinant COVID‐19 vaccines, mucosal IgA‐mediated antibody

## Abstract

**Background:**

The efficacy of immunization against an airborne pathogen depends in part on its ability to induce antibodies at the major entry site of the virus, the mucosa. Recent studies have revealed that mucosal immunity is poorly activated after vaccination with messenger RNA vaccines, thus failing in blocking virus acquisition upon its site of initial exposure. Little information is available about the induction of mucosal immunity by inactivated and recombinant coronavirus disease 2019 (COVID‐19) vaccines. This study aims to investigate this topic.

**Methods:**

Saliva and plasma samples from 440 healthy Congolese were collected including (1) fully vaccinated 2 month postvaccination with either an inactivated or a recombinant COVID‐19 vaccine and (2) nonvaccinated control group. Total anti‐severe acute respiratory syndrome coronavirus 2 receptor‐binding domain IgG and IgA antibodies were assessed using in‐house enzyme‐linked immunosorbent assays for both specimens.

**Findings:**

Altogether, the positivity of IgG was significantly higher in plasma than in saliva samples both in vaccinated and nonvaccinated control groups. Inversely, IgA positivity was slightly higher in saliva than in plasma of vaccinated group. The overall IgG and IgA levels were respectively over 10^3^ and 14 times lower in saliva than in plasma samples. We found a strong positive correlation between IgG in saliva and plasma also between IgA in both specimens (*r* = .70 for IgG and *r* = .52 for IgA). Interestingly, contrary to IgG, the level of salivary IgA was not different between seropositive control group and seropositive vaccinated group. No significant difference was observed between recombinant and inactivated COVID‐19 vaccines in total IgG and IgA antibody concentration release 2 months postvaccination both in plasma and saliva.

**Conclusion:**

Inactivated and recombinant COVID‐19 vaccines in use in the Republic of Congo poorly activated mucosal IgA‐mediated antibody response 2 months postvaccination.

## Graphical abstract

Saliva as a tool for immunological investigation. Poor‐mediated antibody response after COVID‐19 vaccination in Congo.

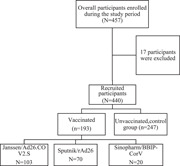

## 1 INTRODUCTION

The worldwide impact of the coronavirus disease 2019 (COVID‐19) pandemic has called for the rapid development of public health measures to control the spread of the virus.^1^ In some ways, however, the virus is under better control since the first cases were identified in Wuhan, China, on December 2019 due to different fighting deployed measures including vaccination.^2^ Regardless of the benefits of those COVID‐19 vaccines in curbing the pandemic, there are still some concerns about the patterns underlying the protection conferred by anti‐severe acute respiratory syndrome coronavirus 2 (SARS‐CoV‐2) antibodies following vaccination, as vaccinated individuals continue to be infected. One of the growing reasons is that mucosal immunity is not considered sufficiently in the design of those vaccines.^3^, ^4^, ^5^, ^6^

Current evidence suggests that mucosal immunity plays an important role in protecting against most respiratory infections including influenza,^7^ poliovirus,^8^ and SARS‐CoV‐2,^6^, ^9^ as it involves the activation of immune cells and secretion of antibodies directed towards mucosal tissues of the respiratory tract, which, in case of SARS‐COV‐2, can help to prevent the virus spike protein to fix in the ACE2 receptor and invade host cells.^6^

Secretory immunoglobins A (sIgA) are the most abundant antibody isotype found in mucosal secretions, such as saliva and mucus. It is produced by plasma cells located in the mucosal‐associated lymphoid tissue and is transported across mucosal surfaces to impede viral replication and shedding in the airways.^6^, ^10^ Additionally, total anti‐SARS‐CoV‐2 IgA, IgM, and IgG in plasma/serum and saliva have been reported during natural infection with an increase of IgA during the early phase of SARS‐CoV‐2 infection.^11^, ^12^, ^13^ With regard to vaccine‐induced antibodies, recent studies have shown that messenger RNA (mRNA) SARS‐CoV‐2 vaccines induce a weak mucosal IgA‐mediated antibody response in saliva and nasal fluid specimens thus failing in limiting virus acquisition upon its entry through this route.^3^, ^4^, ^5^, ^14^

Recombinant vaccines (Sputnik/rAd26, Janssen/Ad26.COV2.S) and inactivated vaccines (Sinopharm/BBIP‐CorV) are the most distributed COVID‐19 vaccines so far (SITREP 208, SITREP 244) in the Republic of Congo (RoC). Clinical trials and real‐world data support the effectiveness of those vaccines in interrupting the chain of transmission and significantly reducing the mortality rate associated with the infection.^15^, ^16^, ^17^, ^18^, ^19^ However, little is known about the mucosal immunity induced by the aforementioned vaccines. The objective of the current study is to assess anti‐receptor‐binding domain (RBD) antibody IgG and IgA in the saliva and plasma following Sinopharm/BBIP‐CorV, Janssen/Ad26.COV2.S, and Sputnik/rAd26 vaccination in Congolese participants.

## 2 METHODS

### 2.1 Study design and participants

The study has been conducted from May to August 2022 in Brazzaville (capital of the RoC), to assess both prevalence and level of mucosal and serum antibodies in the population. A call for participation in the study was launched and interested people aged more than 18 years old were welcomed at the health facility of the Congolese foundation for medical research (Brazzaville) for enrollment if they meet the inclusion criteria.

1) To be healthy confirmed by the clinician after the clinical check‐up
2) To be tested negative by reverse‐transcription polymerase chain reaction (RT‐PCR)
3) To have been fully vaccinated and only a minimum of 2 months postvaccination participants were enrolled in this study including BBIP‐CorV (Sinopharm 2×) or Janssen/Ad26.COV2.S (Johnson & Johnson 1×), or rAd26 (Sputnik 2×) vaccines.
4) A nonvaccinated control group including individuals with no history of prior SARS‐CoV‐2 infection. The study period experienced a high level of vaccine hesitancy, resulting in many enrolled individuals remaining unvaccinated. These individuals met the inclusion criteria of testing negative for SARS‐CoV‐2 at the time of enrollment and reporting no prior positive results from RT‐PCR testing.

Informed consent was given before enrollment and after clinical examination, the clinician recorded socio‐demographic and clinical data and vaccine description (brand, type, date of vaccination). The study clinical protocol was approved by the Institutional Ethics Committee of the Congolese Foundation for Medical Research (038/CIE/FCRM/2022).

### 2.2 Treatment of samples

Oropharyngeal swabs was performed for SARS‐CoV‐2 infection screening by PCR using the QIAamp Viral RNA Mini Kit (Qiagen) for RNA extraction and subjected to RealStar® SARS‐CoV‐2 (Altona Diagnostics) for RNA detection. Positive individuals were excluded from the final analysis.

From each negative participant, blood (5 mL) was collected using lithium heparin monovets or EDTA and obtained plasma was stored at −20°C. Saliva was collected by spitting into a simple plastic tube (multipurpose containers 50 mL Greiner Bio‐One ref. 201150). Before sample collection, participants were asked to refrain from consuming food, drink, or tobacco products for at least 30 min. Each enrolled subject underwent self‐collection of whole saliva samples under the supervision of a trained healthcare professional. Participants were instructed not to swallow saliva for at least 30 s to 1 min, to accumulate a significant amount before spitting into a sterile container. Saliva samples were immediately transferred in two (2) 1.5 or 2 mL reaction tubes and kept at −20°C. Twenty‐five additional plasma samples from archived prepandemic period (before 2019) were used as negative control.

### 2.3 Measurement of antigen‐specific SARS‐CoV‐2 IgG in saliva and plasma

In‐house enzyme‐linked immunosorbent assays (ELISAs) specifically established to analyze IgG antibodies in plasma and saliva, reacting against the SARS‐CoV‐2 RBD of the ancestral strain were used.^20^ Briefly, high‐binding plates (Corning, ref: 3590) were coated overnight with 50 µl per well of 2 µg/mL of the SARS‐CoV‐2 RBD antigen suspended 1× phosphate‐buffered saline (PBS) (Gibco, ref: 18912‐014). Wells were washed once with 1× PBS without detergent, 200 µL per well, and blocked with 100 µL of The Blocking Solution (Candor Bioscience GmbH) for 2 h at room temperature (RT) on a microplate shaker (700 rpm). Plasma samples of participants and 25 archived prepandemic plasma were serially diluted from 1:100 to 1:62,500 using the blocking solution. The plates were washed three times with 200 µL per well of × PBS + 0.1% Tween 20. Then, 100 mL of sample dilution was added per well and incubated for 1 h (RT, 700 rpm). After plate washing, 50 µL per well of an horseradish peroxidase (HRP)‐coupled antihuman IgG was used (Jackson Immuno Research Laboratories, ref. 109‐036‐097). The detection antibody was diluted 1:10.000 in 1× ROTI Block buffer (Carl Roth, ref. A151.2) and incubated for 30 min (RT, 700 rpm). For visualization, 100 µL TMB substrate solution was added after four times washing and the reaction was stopped using 1 M HCl. Plates were measured at 450 nm and 620 nm with a microplate reader (CLARIOstar, BMG LABTECH). The IgG concentration was presented in µg/mL and the cut‐off value was previously set to 4.0 µg/mL.^20^ The SARS‐CoV‐2 IgG detection in saliva was performed following similar parameters as used in the plasma ELISA assay, with some exceptions: Saliva was diluted from 1:3 up to 243 using the same blocking solution. For IgG detection, a 1:20,000 in 1× ROTI Block diluted biotinylated antihuman IgG was incubated for 1 h and 1:1000 Avidin‐HRP (Biolegend ref. 405103) was applied for 30 min. The cutoff for salivary SARS‐CoV‐2 IgG positivity was previously set to 6.3 ng/mL.^20^

### 2.4 Measurement of antigen‐specific SARS‐CoV‐2 IgA in saliva and plasma

An in‐house ELISA specifically established to analyze IgA in plasma and saliva reacting to SARS‐CoV‐2 RBD of the ancestral strain were used.^20^ The same parameters for IgG were also used here with some exceptions: High binding plates were coated overnight with 50 µL per well of 1 µg/mL of the SARS‐CoV‐2 RBD antigen for IgA in plasma and 2 µg/mL in saliva. Wells were blocked with 200 mL of The Blocking Solution (Candor) for IgA plasma and 10% of milk prepared in 1× PBS with 0.1% Tween 20 for saliva (incubated for 2 h at RT). Plasma samples and 25 archived prepandemic plasma were serially diluted from 1:100 to 1:12,500 using the blocking solution. For Plasma IgA detection, an HRP‐coupled antihuman IgA was used (Jackson LOT#109‐035‐011). The detection antibody was diluted 1:5000 in 1× ROTI Block buffer and incubated for 30 min (RT, 700 rpm). The cutoff for plasma SARS‐CoV‐2 IgA positivity was previously set to 0.5 µg/mL.^20^ Saliva samples and controls were serially diluted from 1:3 to up to 1:81 using the 10% milk and for salivary IgA detection a 1:10,000 diluted biotinylated antihuman IgA (in milk) and 1:1000 Avidin‐HRP in 1× Roti were used.

**Figure 1:**
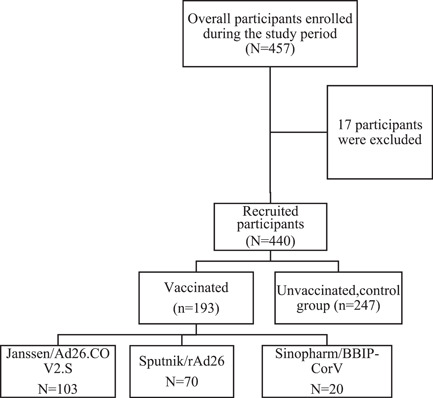
Receiver operating characteristic curve (ROC curve) for salivary IgA cutoff determination.

To estimate IgA threshold point in saliva, we computed the receiver operating characteristic curve (ROC) curve analysis as previously described obtaining an ROC curve of 94.66% (95% confidence interval [95% CI]: 89.65%–99.66%) and a combination of specificity and sensitivity metrics for different cut‐off values. We then selected as optimal threshold of 28.69 ng/mL, which featured a sensitivity of 69.14% (95% CI: 61.65%–75.74%), and a specificity of 97.14% (95%CI: 85.47%–99.85%) (Figure 1).

A cutoff for IgG in plasma/saliva and IgA in plasma was already available as described by.^20^ We have confirmed their effectiveness by using pre‐pandemic samples and finding all samples being negatives for IgA (0 positive out of the 25 samples) and one sample (1/25) unfortunately barely crossing the cut‐off line for IgG (Figures 4B and 6B).

### 2.5 Statistical analysis

RStudio (Version 1.2.5001), running R (version 4.2.1.), and Graph Pad Prism (Version 8.0.2) were used for statistical analyses. The significance was defined by a 95% confidence interval (*p* < .05). Categorical variables were reported as percentages and compared using Fisher's exact test. Continuous variables were reported as median (interquartile range, IQR). The difference in antibody levels between two groups was analyzed with Mann–Whitney test, whereas the Kruskal–Wallis test followed by Dunn's multiple comparison test was used for comparing more than two groups. Correlations between antibody concentrations were performed using Pearson's test.

## 3 RESULTS

### 3.1 Characteristics of the participants

**Figure 2:**
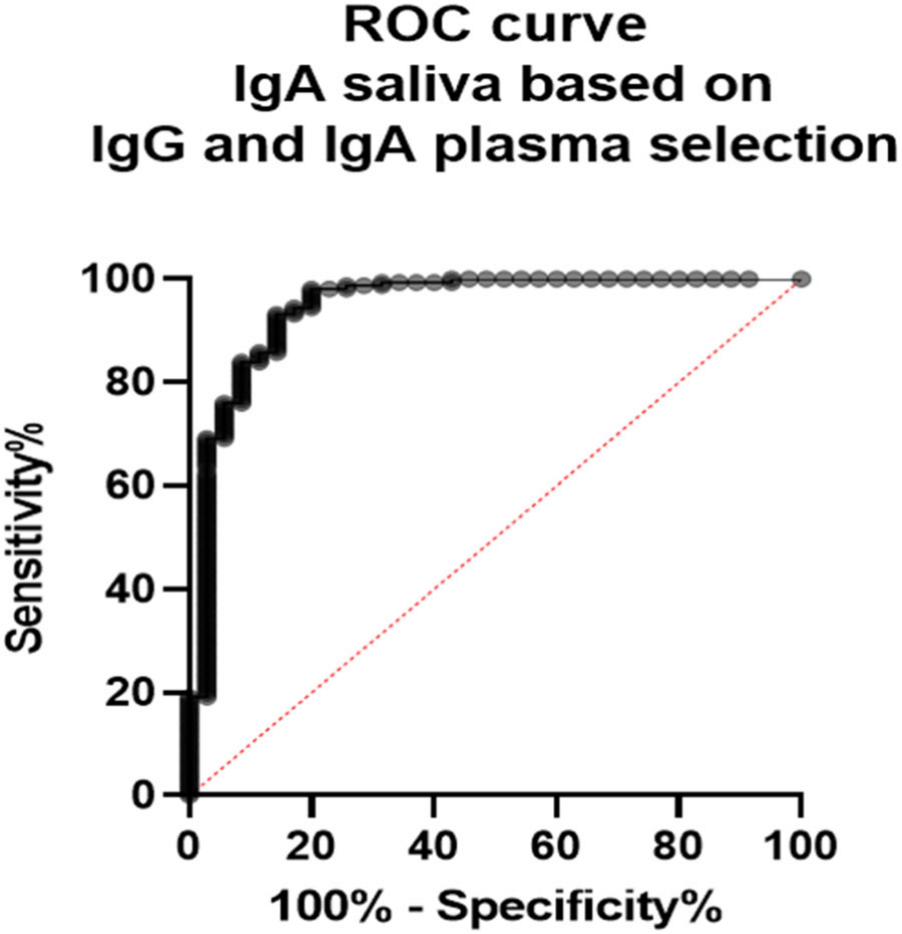
Recruitment of participants.

A total of 440 participants were recruited including 193 vaccinated and 247 nonvaccinated control group (Figure 2). Males were significantly more represented than female (Fisher's exact test: *p* = .0160). The median age of the participants was 27 years (ranging from 18 to 83). The demographic characteristics of the included participants are shown is the Table 1.

**Table 1:** Characteristics of study population.

	Number	Frequency (%)
Overall population	440	–
Sex		
Female	180	41
Male	260	59
Median age (range)	27 (18; 83)	
Vaccine status		
Vaccinated with (dose)	Ad26.COV2.S: 103 (×1)	43.9
	rAd26: 70 (×2)	
	BBIP‐CorV: 20 (×2)	
Nonvaccinated	247	56.1

### 3.2 Total anti‐SARS‐COV‐2 IgG positivity in plasma and saliva samples in vaccinated participants

**Figure 3:**
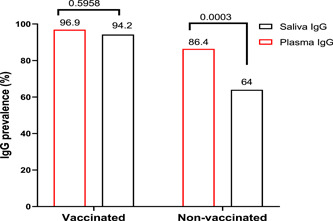
Seroprevalence of anti‐severe acute respiratory syndrome coronavirus 2 IgG in plasma and saliva by vaccinated group and nonvaccinated control group. Fisher's exact test was used for the comparison of IgG seroprevalence between saliva and plasma among groups.

The overall result revealed that the estimated IgG positivity in non‐vaccinated control group was significantly higher in plasma (211 positive out of the 244; 86.4%) than in saliva (151 positive out of the 237; 64%; Fisher's exact test: *p* = .0003). The same was observed in vaccinated group with 96.9% (186/192) of the positivity in plasma versus 94.2% (179/190) in saliva but no significantly *p* = .5958 (Figure 3).

### 3.3 Total anti‐SARS‐COV‐2 IgG level in plasma and saliva samples in vaccinated participants

**Figure 4:**
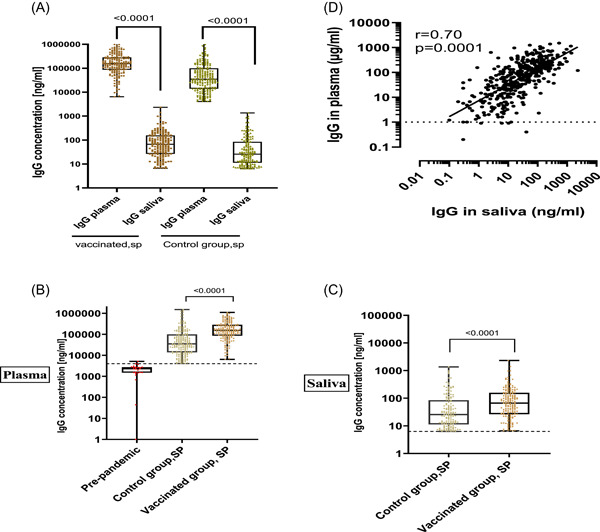
Level of severe acute respiratory syndrome coronavirus 2‐specific total IgG in plasma and saliva of seropositives (SPs). (A) Level of total IgG antibody in plasma versus saliva specimens of vaccinated and control group. (B) Level of total IgG in plasma of SP vaccinated group (*n* = 186) versus of nonvaccinated SP group (*n* = 211) and prepandemic (*n* = 25). (C) Level of total IgG in saliva of SP vaccinated (*n* = 179) versus nonvaccinated SP group (*n* = 151). (D) IgG Pearson correlation between saliva and plasma. The horizontal lines show the median values, the box plots show the interquartile range and the whiskers indicate the minimum‐to‐maximum range. Each dot corresponds to an individual subject. *p* values were determined using the Mann–Whitney.

We assessed and compared the magnitude of the total IgG response of seropositive (SP) individuals according to the specimen and groups. The results revealed that the median IgG concentration was over 1000‐fold higher in plasma than in saliva of the SP nonvaccinated (control group, SP) *p* = .0001. The same was observed in SP vaccinated group (vaccinated, SP) *p* = .0001 (Figure 4A). When comparing IgG antibody level between saliva of vaccinated, SP, and saliva of the control group, SP results showed that vaccinated individuals harbored significantly higher IgG level than control group *p* = .0001. The same was found in plasma (Figure 4B,C and Table 2). Correlations analysis further indicated that the level of salivary IgG of the participants positively correlated with that of plasma IgG antibody (*r* = .70, *p* = .0001) (Figure 4D).

**Table 2:** Summary of the relevant comparison on the magnitude of the total IgG and IgA response in plasma and saliva of positive (SP) participants.

Variables	Overall	Vaccinated, SP	Control group, SP	*p* a	*p* b
Plasma IgG concentration in ng/mL, median (IQR)	84,254 (26,927; 190,052)	154,450 (85,602; 284,132)	34,894 (13,849; 99,225)	.0001	.0001
Saliva IgG concentration in ng/mL, median (IQR)	42.80 (15.00;124.0)	66.80 (26.50;156.8)	25.80 (11.30; 84.8)	.0001	.0001
Plasma IgA concentration ng/mL, median (IQR)	1055 (731.9;1846)	1096 (761.1; 1846)	1028 (642.2; 1834)	.8673	.0001
Saliva IgA concentration in ng/mL	70.75 (42.63;111.7)	78.54 (39.22; 111.7)	61.77 (45.22; 112)	.5599	.0001

Abbreviations: IQR, interquartile range; SP, seropositive.

^a^ Testing the median values in IgG level between plasma of vaccinated, SP vs. plasma of control group, SP and between saliva of vaccinated, SP vs. saliva of control group, SP. The same was done with IgA.

^b^ Testing the median values of IgG and IgA between samples types in groups (plasma IgG vs. salivary IgG in vaccinated, SP; plasma IgA vs. salivary IgA in vaccinated, SP). The same was done in nonvaccinated group.

### 3.4 Total anti‐SARS‐COV‐2 IgA positivity in plasma and saliva samples in vaccinated participants

**Figure 5:**
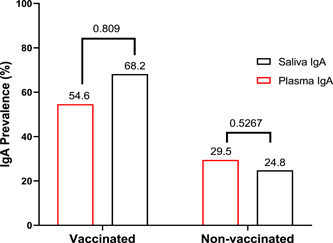
Seroprevalence of anti‐severe acute respiratory syndrome coronavirus 2 IgA in plasma and saliva by vaccinated group and nonvaccinated control group. Fisher's exact test was used for the comparison of IgA seroprevalence between saliva and plasma among groups.

The estimated IgA positivity in nonvaccinated control group was higher in plasma (72 positive out of the 244; 29.5%) than in saliva with no significance (51 positive out of the 205; 24.8%; Fisher's exact test: *p* = .5267). However, not the same was observed in vaccinated group where we found IgA positivity being slightly higher in the saliva (127 positive out of the 186; 68.2%) than in the plasma (105 positive out of the 192; 54.6%; Fisher's exact test: *p* = .809) with no significance (Figure 5).

### 3.5 Total anti‐SARS‐COV‐2 IgA levels in plasma and saliva samples in vaccinated participants

**Figure 6:**
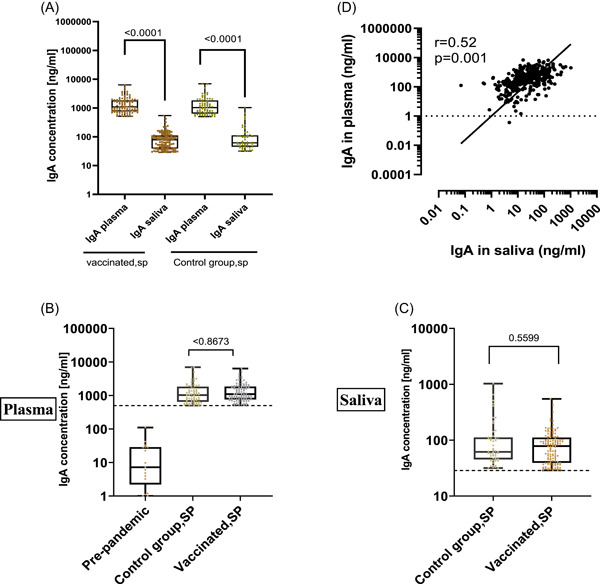
Level of severe acute respiratory syndrome coronavirus 2‐specific total IgA in plasma and saliva of seropositive (SP). (A) Level of total IgA antibody in plasma versus saliva specimens of vaccinated and control group. (B) Level of total IgA in plasma of SP vaccinated group (*n* = 105) versus of nonvaccinated SP group (*n* = 72) and prepandemic (*n* = 17). (C) Level of total IgA in saliva of SP vaccinated (*n* = 127) versus nonvaccinated SP group (*n* = 51). (D) IgA Pearson correlation between saliva and plasma. The horizontal lines show the median values, the box plots show the interquartile range and the whiskers indicate the minimum‐to‐maximum range. Each dot corresponds to an individual subject. *p* values were determined using the Mann–Whitney.

When assessing and comparing the magnitude of the total IgA response of SP individuals according to the specimen and group, the results revealed that the median IgA level was about over 14‐fold higher in plasma than in saliva of the SP nonvaccinated group (control group, SP) *p* = .0001. The same was observed in SP vaccinated group (vaccinated, SP) *p* = .0001 (Figure 6A). We found the similar IgA antibody level between saliva of vaccinated and saliva of the control group *p* = .5599. The same observation was made in plasma sample *p* = .8673 (Figure 6B,C and Table 2). Correlation analysis further indicated that the level of salivary IgA of the participants positively correlated with that of plasma (*r* = .52, *p* = .0001) (Figure 6D).

### 3.6 Assessment of total IgA and IgG level in saliva and plasma according to the type of vaccine

**Figure 7:**
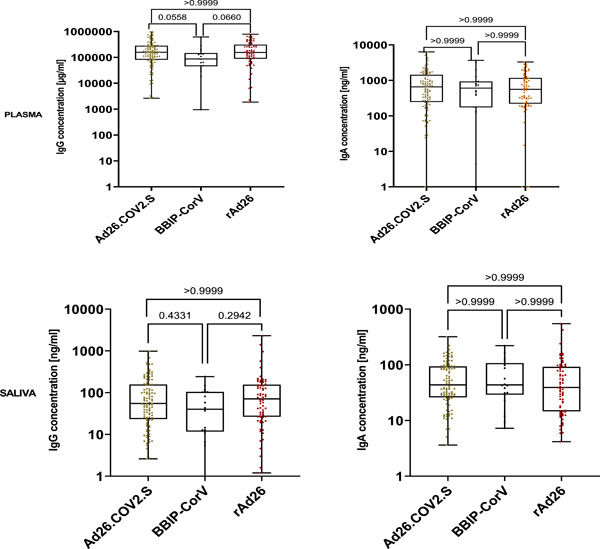
Median IgG and IgA severe acute respiratory syndrome coronavirus 2 antibody concentration release in plasma and saliva after vaccination with Ad26.COV2.S, rAd26, and BBIP‐CorV vaccines. The horizontal lines show the median values, each dot corresponds to an individual subject. *p* values were determined using the Kruskal–Wallis test with the Dunn's multiple comparison test.

When evaluating antibody response according to the type of the vaccine received, results indicate that the three vaccines (Ad26.COV2.S, rAd26, and BBIP‐CorV vaccines) induced IgG and IgA level with no differences both in plasma and saliva (Figure 7 and Table 3).

**Table 3:** Comparison of the median IgG and IgA SARS‐CoV‐2 antibody release between Sputnik/rAd26, Janssen/Ad26.COV2.S, and Sinopharm/BBIP‐CorV COVID‐19 vaccine.

Variables	Ad26.COV2.S	rAd26	BBIP‐CorV	*p*
Plasma IgG concentration in ng/mL Median (IQR)	157895 (815110; 283801)	155054 (88584; 311820)	86735 (45428;146997)	.0516
Saliva IgG concentration in ng/mL Median (IQR)	55.20 (23.30;157.6)	71.10 (26.40;155.8)	40.10 (11.70; 104.8)	.2506
Plasma IgA concentration ng/mL Median (IQR)	657.4 (246.2;1444)	560.2 (220.7; 1173)	605.9 (173.7; 932.6)	.8878
Saliva IgA concentration in ng/mL Median (IQR)	43.57 (25.98;93.95)	39.15 (14.62; 91.99)	43.66 (29.10; 107.4)	.5388

Abbreviations: COVID‐19, coronavirus disease 2019; IQR, interquartile range; SARS‐Cov‐2, severe acute respiratory syndrome coronavirus 2.

## 4 DISCUSSION

Understanding the magnitude and compartmentalization of antibody in a diverse population is essential in designing mass vaccination strategies taking into account the type of vaccine to use and its effectiveness. We evaluated the magnitude of salivary antibody release after vaccination with either an inactivated (BBIP‐CorV) or a recombinant (rAd26 and Ad26.COV2.S) COVID‐19 vaccine in use in the RoC during the study period in comparison with nonvaccinated control group.

We found that SARS‐CoV‐2 IgG positivity was higher in plasma than in saliva in both vaccinated and non‐vaccinated control group. This could be explained by the fact that most of the salivary IgG antibodies likely originate in the plasma by transudation from the bloodstream.^21^ To further clarify this point, we assessed the magnitude of the total anti‐SARS‐CoV‐2 IgG antibodies in saliva and plasma then processed multiple comparisons and correlation analyses. We found a median concentration of IgG approximately a 1000 times higher in plasma than in the saliva. We also found a high positive correlation between salivary IgG and plasma IgG, supporting the hypothesis of a plasma derived origin of salivary IgG.^21^, ^22^ When comparing the IgG antibody magnitude in saliva samples of positive groups (i.e., saliva of positive vaccinated vs. saliva of positive control group), we found vaccinated displaying a significantly higher level of IgG than of the control group. The same was observed in plasma. An explanation could be the potent immunogenic properties of the administered COVID‐19 vaccines, as previously reported.^15^, ^16^, ^18^, ^23^ Furthermore, the robustness of the IgG antibody response in naturally exposed (here positive control group) individuals has been reported to vary depending on the severity of symptoms (asymptomatic, mild symptomatic, and highly symptomatic).^24^

In contrast to IgG, we found that the overall IgA positivity was slightly higher in saliva than in plasma of vaccinated. At first sight, this means that vaccination has induced IgA released at plasma and saliva site. It is well known that the IgA in saliva has a dual origin depending on the isotype (sIgA and monomeric [mIgA])^25^: The sIgA has been reported to be locally generated, whereas the mIgA has been reported to be at 77% a plasma‐derived IgA.^22^, ^25^, ^26^, ^27^, ^28^ Together, this could explain why the IgA positivity was a bit higher in saliva than in plasma. To further bring light in this point, we assessed the magnitude of the total anti‐SARS‐CoV‐2 IgA in saliva and plasma of the overall participants then processed multiple comparisons and correlation analysis. We found a median concentration of IgA ~14‐fold higher in plasma of participants than in saliva. We also found a high positive correlation between salivary IgA and plasma IgA (*r* = .52). These findings suggest that the majority of salivary IgA evidenced here was a plasma‐derived isotype (mIgA).^21^ Further analysis would better confirm this. When comparing the IgA antibody magnitude in saliva samples of positive groups (i.e., saliva of positive vaccinated vs. saliva of positive control group), interestingly, we found the similar IgA antibody level between both groups. The same was observed in plasma. This result suggest that the COVID‐19 vaccines used in the RoC during the study period would not have induced a robust mucosal response associated with IgA. Similar results were recently reported for people vaccinated with mRNA COVID‐19 vaccines,^3^, ^4^, ^5^, ^29^ indicating that COVID‐19 vaccines in general do not induce a robust mucosal IgA associated antibody response, which may explain the reinfection seen worldwide among vaccinated.^30^

The analysis of the total amount of IgG and IgA released after vaccination with Recombinant vaccines (Sputnik/rAd26, Janssen/Ad26.COV2.S) and inactivated vaccine (Sinopharm/BBIP‐CorV), both in saliva and plasma samples, show no difference, indicating that all those vaccines were equally immunogenic.

The overall high IgG prevalence in non‐vaccinated control group in this study indicates that the population of Brazzaville, the epicenter of the pandemic in the RoC, already achieved herd immunity against SARS‐COV‐2. This is supported by a low PCR positivity rate reported in the national situation report (SITREP) during the study period (turning around 2%) [SITREP 208‐244] and, also by considering our previous findings.^31^, ^32^ Nevertheless, the state of herd immunity is probable to be bypassed by other viral variants, which are less efficiently blocked by the antibodies generated in previous infections.^33^, ^34^

## 5 CONCLUSION

Inactivated and recombinant COVID‐19 vaccines in use in the RoC would not have induced a robust mucosal response associated with IgA.

## AUTHOR CONTRIBUTIONS

**Freisnel H. Mouzinga**: Conceptualization, investigation, methodology, writing of original draft, data analysis, review, and editing. **Constanze Heinzel**: Sample processing or methodology, review, and editing, **Abel Lissom**: Investigation, review, and editing. **Armel L. Batchi‐Bouyou**: sample collection, review, and editing. **Jean C. Djontu**: Review and editing. **Jacques D. Mbama Ntabi**: Sample collection. **Etienne Ngumbi**: Review and editing. **Francine Ntoumi**: Conceptualization, funding acquisition, investigation, methodology, project administration, supervision, validation, visualization, review, and editing. **Rolf Fendel**: Funding acquisition, investigation, methodology, supervision, validation, visualization, review, and editing. **Peter G. Kremsner**: Head of the ITM laboratory, review, and editing. All authors read and approved the final manuscript. **Andrea Kreidenweiss**: Investigation, methodology.

## CONFLICT OF INTEREST STATEMENT

The authors declare no conflict of interest.

## ETHICS STATEMENT

This study received ethical approval from the independent Institutional Ethics Committee of Fondation Congolaise pour la Recherche Medical Avis No. 038/CIE/FCRM/2022) administrative authorizations from Marien Ngouabi University (No. 081/UMNG. FST.DFD.FD‐SBIO) and the administrative authorizations of the Goma Tsé‐Tsé district sub‐prefecture (No. 003/MATDDUDP/DGTT/SG‐02). All participants gave written informed consent to participate in the study.

## Data Availability

All data are fully available upon request. Data are available from the FCRM Institutional Data Access. All request for Data should be addressed to the Executive Director of FCRM, Villa D6, Cité OMS Djoué, Brazzaville, République du Congo (Tel. +242 06 9977980, email: info@fcrm-congo.com.
